# An Approach for 3D Modeling of the Regular Relief Surface Topography Formed by a Ball Burnishing Process Using 2D Images and Measured Profilograms

**DOI:** 10.3390/s23135801

**Published:** 2023-06-21

**Authors:** Stoyan Slavov, Lyubomir Si Bao Van, Diyan Dimitrov, Boris Nikolov

**Affiliations:** 1 Department of Mechanical Engineering and Machine Tools, Technical University of Varna, 9010 Varna, Bulgaria; lubomir.van@tu-varna.bg; 2 Department of Mechanics and Machine Elements, Technical University of Varna, 9010 Varna, Bulgaria; dm_dimitrov@tu-varna.bg; 3 Department of Communication Engineering and Technologies, Technical University of Varna, 9010 Varna, Bulgaria; boris.nikolov@tu-varna.bg

**Keywords:** regular reliefs, ball burnishing, three-dimensional surface topography, signal correlation, signal similarity assessment, topography assessment criteria

## Abstract

Advanced in the present paper is an innovative approach for three-dimensional modeling of the regular relief topography formed via a ball burnishing process. The proposed methodology involves capturing a greyscale image of and profile measuring the surface topography in two perpendicular directions using a stylus method. A specially developed algorithm further identifies the best match between the measured profile segment and a row or column from the captured topography image by carrying out a signal correlation assessment based on an appropriate similarity metric. To ensure accurate scaling, the image pixel grey levels are scaled with a factor calculated as being the larger ratio between the ultimate heights of the measured profilograms and the more perfectly matched image row/column. Nine different similarity metrics were tested to determine the best performing model. The developed approach was evaluated for eight distinct types of fully and partially regular reliefs, and the results reveal that the best-scaled 3D topography models are produced for the fully regular reliefs with much greater heights. Following a thorough analysis of the results obtained, at the end of the paper, we draw some conclusions and discuss potential future work.

## 1. Introduction

Mechanical processing of machine parts is a crucial aspect in various industries, including machine building, aircraft and shipbuilding, automotive manufacturing, etc., and it involves designing and conducting operations that are likely to have a profound impact on the quality characteristics of the machine parts’ contact surfaces, with an immediate effect on the operational performance of the corresponding units as a result. The accuracy of dimensions and shape, along with the quality of the mechanical surfaces, is largely dependent on the resultant surface topography. Applying traditional mechanical finishing operations, such as finishing milling, turning, grinding, etc., produces noticeable changes in the quality of the surface topography, which is normally assessed based on the roughness obtained. International standards for surface texture description, such as ISO 21920-2:2022 (profile) [1] and ISO 25178-2:2021 (areal) [2], prescribe a full range of roughness criteria that can be used to describe the characteristics of the mechanical surface topography. The profile topography criteria defined in ISO 21920 is generally suitable for most mechanical surfaces obtained after the finishing process, given the uniform, albeit stochastic, distribution of their roughness. Attaining close values for the roughness criteria, thus, pre-supposes the inconsequentiality of the precise section of the machined surface within which the topographic profile will be measured.

**Figure 1:**
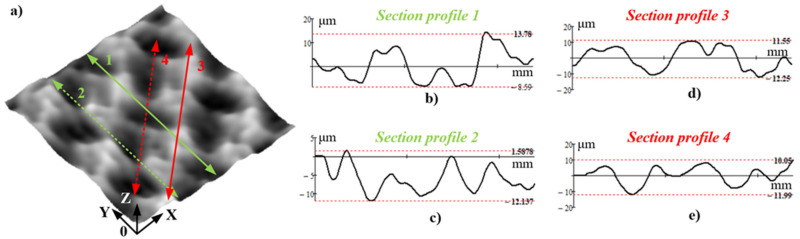
Gaussian filtered view of a regular relief’s three-dimensional topography from the IV-th type, obtained after BB-operation: (**a**) Four random directions for measuring section profiles; (**b**–**e**) resulting topography section profiles in corresponding direction (dashed red lines visualize the ultimate heights of each measured section profiles).

Mechanical surfaces, however, subject to some finishing operations, will easily acquire radically different topography characteristics when compared to traditional surfaces. For instance, the so-called “regular reliefs” (RR), which are produced through the process of ball-burnishing as a finishing operation, are included in that category. Ball burnishing (BB) is a finishing process in which a hard ball of known diameter is pressed firmly against the machined surface, causing plastic deformation (when cold) to occur in the surface layer of the material [3]. When the deform tool follows a complex toolpath trajectory, which occurs as it moves during the BB-operation performed on a CNC lathe or milling machines [4,5], specific RR patterns are imprinted onto the burnished surface. As can be seen in Figure 1a, the areal topography of fully-RR BB-operation is a pattern of comparatively regularly spaced “dimples”, which are created upon the crossing of the plastically deformed traces inscribed using the deform tool.

According to the RR classification [3,6] there are five distinct types of regular reliefs, with the first three of them pertaining to partially regular reliefs, while the last two types refer to fully regular reliefs. A “fully regular relief” means that the plastically deformed traces, which are left behind by the deform tool, cover the burnished surface completely without any remaining “islands” with residual topography from the preceding cutting operation, as opposed to the partially-RR. Each of the five types of RR could be imprinted onto the burnished surface relative to the pre-set BB-process regime parameters. When a BB operation is performed via a CNC machine, the shape and size of the RR cells can vary to a considerable extent [7]. Thus, for example, the cells from a fully-RR topographic representation are generally expected to span the range of 15–150 μm (according to Sz-criterion from ISO 25178-2:2021 [2]) with regard to the burnished material characteristics, the deforming ball diameter, and applied deforming force, while the presumed length of the RR cells borders is likely to exceed 1600 μm. These particular parameters, taken together with the comparatively high recurrence rates of the cells in the X-Y plane, help establish the BB-process-based RRs as a geometric deviation based on “waviness” rather than “surface roughness”. Unlike the traditional mechanical textures, the RR textures retain more lubricants and products of wear within their furrowed dimples. The roughness of the plastically deformed surface within the cells is very low, and the radii of the ridges (i.e., the borders of the cells) are much bigger than those obtained via some other well-known finishing operations. The resulting properties make them well suited for application in heavy loaded units, transport systems, and vehicles that are subjected to harsh operating conditions and are equipped with sliding pairs, metal-rubber joints, couplings, sealing systems, etc. 

It is of utmost importance for the RR topographic characteristics to be accurately determined in order to predict their prospective operational behavior or compare them with textures obtained through the adoption of other finishing techniques. Regrettably, the widespread methods for surface topography measurements present an enormous challenge to the RRs. For instance, a single profile, which is measured in a given section, does not give reliable results for the RR topographic criteria, as defined by standards ISO 21920-2:2021 (or former ISO 4287 [8]). This problem occurs because the measured topography profile is conditioned by the section and direction of the stylus of the contact roughness measuring device. Figure 1a depicts four random directions of measuring the surface profile, with the corresponding measured profiles towards these directions being constructed in Figure 1b–e. 

As outlined in Figure 1b–e, the profiles derived in the different sections for the same RR topography display a wide disparity both along the profile and in a vertical direction. Hence, if the RR topographic features are determined via a single profile measurement in only one section, the results obtained, in all probability, will be highly controversial. Using the areal approach, in line with the established criteria set forth in the ISO 25178-2:2021 [2] standard, seems more relevant to the case under study. There are many commercial 3D texture measuring devices available nowadays, which use different approaches for contact and non-contact measuring of the surface topography, which is in agreement with the classification specified in ISO 25178 standard, part 6 [9]. Non-contact measuring devices offer significantly higher accuracy and comparatively faster scanning capabilities compared to their contact-based counterparts [10,11,12,13]. Non-contact methods present certain drawbacks, such as the requirement for a specific level of reflectivity in the scanned texture, the non-transparency of the sample material, uneven measurement step in height, etc.

The relatively high cost of the non-contact measuring devices, which could reach several hundred thousand USD relative to their specific characteristics, remains an almost insurmountable obstacle to their common utilization. Another significant constraint on the use of non-contact devices is their limited scanning area, which is characteristic of the cheaper versions in particular, where the dimensions can be generally as small as a few tens or hundreds of micrometers at the expense of the high scanning resolution in height. Such a limited measurement area, however, cannot accommodate even a single cell of the RR, let alone a larger pattern of RR cells to be scanned in its entirety. Alternatively, contact 3D profilometers offer a more cost-effective solution, albeit with a lower resolution in both lateral and height directions, as well as lower scanning speed.

The focus of research is currently shifted towards the possibility of machining processes that incorporate artificial intelligence (AI) and deep learning (DL) techniques for automation control in manufacturing operations and their output. Computer vision (CV), which is one of the fields of AI [14], involves training computers via specially developed algorithms to interpret data from digital image(s) or video(s) in order to recognize objects, patterns, colors, surface characteristics, and other elements. Over the years, numerous research studies reported results related to the CV techniques and algorithms in mechanical processing of machined parts. For instance, CV is used to perform reverse engineering of machine parts [15,16,17], automatic tool selection in computer numerically controlled (CNC) lathe and milling machines [18,19], calculation of the machining time of cutting tools using machined parts images [20], automatic programing, and control of CNC machines [21,22,23], etc. Computer vision techniques are also used in micro-scale surface texture characterization of machined surfaces [24,25], automotive cylinder liner surfaces [26], surface topography using shape from shading [27], nanoscale measurements of diamond machined polar and mixed microstructures [28,29,30,31], micro vision-based precision motion measurements [32], reconstructing high-resolution images from low-resolution images [33], etc.

Several other specific methods used for 3D reconstruction from 2D images of large-scaled objects can be seen in studies by Schonberger et al. [34], Hongmin et al. [35], Gao et al. [36], Munkberg et al. [37], and Fan et al. [38]. They explore different techniques used to obtain three-dimensional reconstruction of urban and/or indoor scenes, which are based on ordered or disordered multi-image sets. One method is to use multiple cameras to capture the reconstructed object from multiple sides through different angles or a single photo- or video-camera that moves around the object and capture images during a certain time interval are used for that purpose. According to the specificity of RRs, they are not directly applicable to their three-dimensional reconstruction for the following reasons: their purpose is to reconstruct a three-dimensional model of the entire object (building, neighbor-hood, or theater hall), rather than the topography of a single surface; the accuracy of the reconstructed model will be relatively high, albeit only compared to the large scale of the objects; and these methods require a large number of captured object images (sometimes more than 150k images), as well as computers with very large random access memory (RAM) and faster processors to complete calculations in a reasonable time interval.

Developed in several research works is the conclusive argument that the long-established approaches [39,40,41] for surface topography recognition could be applied not only for micro- and nanoscale surface textures, but also for the identification of larger-scale textures, such as the RRs discussed above. Given that the scale of a RR is much larger than that of a diamond machined microstructure, it is reasonable to assume that many of the distracting factors influencing the results of micro- and nano-scale measurements will be less pronounced in such a context. This fact will enable the application of 2D images with moderate resolution and cost-effective measuring equipment for the digital recreation of a RR’s topography. The primary objective of the current paper, therefore, is to examine the feasibility of developing a technique for rapid three-dimensional RR topography modeling with the help of two-dimensional greyscale images and profilograms measured via a roughness tester with a stylus.

## 2. Materials and Methods

The proposed methodology was based on the approach outlined in [24], which applied a machine vision technique to contrast two-dimensional grayscale images with stylus-measured profiles in order to provide a careful assessment of the roughness of mechanically processed surfaces. Obtaining a three-dimensional representation of the RR’s topography was accomplished through three main stages. The first stage involved obtaining the appropriate two-dimensional RR image and its subsequent filtering to compensate for the concomitant image distortions and noise. Identified, at this stage, was the image row and the column with the maximum difference between the highest and lowest values of the pixel grey levels. Employed, in the second stage, was a stylus profilometer to measure the actual RR profile heights in two perpendicular sections, acting as close as possible to the previously identified rows and columns with maximum grey level differences. However, due to the intrinsic difficulty in synchronizing the identified row and column from the RMR image and the profile-measured section, another algorithm was developed to establish the highest level of correlation between the image rows/columns and the real profiles from the measured RR. Once the highest correlation level was established, the scaling factor was calculated as the ratio between the measured profile height and the difference between the highest and lowest pixel grey level values of the row/column. In the final step, the remaining rows/columns from the topography image (TI) were scale limited with the obtained scaling factor. A detailed explanation of the proposed approach, which was based on Python programing language and corresponding libraries, is provided below. 

### 2.1. Methodology for Obtaining a Two-Dimensional Image of the RR Topography

**Figure 2:**
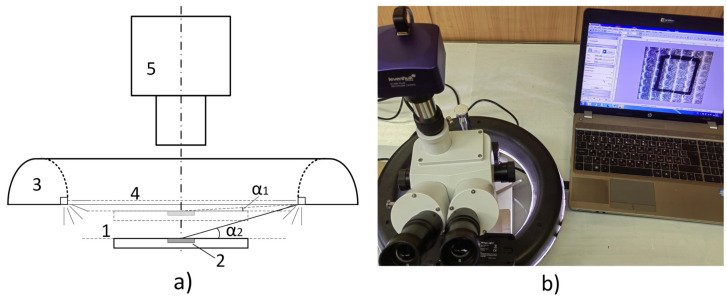
Diagram (**a**) and picture (**b**) of RR’s topography imaging setup, where: 1—planar specimen with RR texture; 2—photographed area; 3—circular diffuse lamp; 4—light emitting plane; 5—high-resolution 4 k CMOS camera, mounted on microscope’s trinocular head; α1—high and α2—low angle between planar specimen with RR and light emitting plane of halo lamp.

A square area from the RR topography was photographed using trinocular head microscope and 14-megapixel CMOS digital camera with high resolution (see Figure 2a,b). To capture an accurate surface image, it was essential to use a diffuse light source that emitted equalized light from all directions, which was compatible with the pattern being discussed. A diffuse circular video lamp (or so-called “halo-lamp“) was considered quite appropriate for that purpose as it is immovably fixed. To illuminate effectively the RR patterns, it was necessary to ensure that the angle of the light source and the pattern plane is sufficiently low to simulate the conditions of a sunrise or sunset in the mountains, where the peaks are brightly illuminated and the valleys remain dark. This approach minimized the presence of glare from the metallic smooth inner surfaces of the RR cells in the CMOS sensor and helped achieve a clear-cut distinction between the peaks and the valleys. 

The values of α (the angle of light) were limited by the dimensions of the halo lamp and the microscope. The angle measurement was performed via simple trigonometric calculations, such as measuring the inner diameter of the circular lamp and the distance between the light emitting plane and the top surface of the RR specimen. The angular measurement of the light direction was selected as a method to facilitate further redesign of the experiment should other equipment with distinctive design and/or dimensions be used.

**Figure 3:**
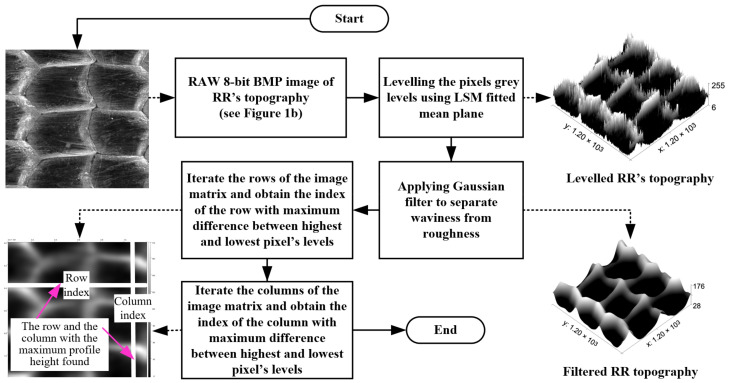
An algorithm for initial processing of BMP image that represents RR topography.

The pictures obtained in this way were recorded as raster graphic images in bitmap (BMP) files with resolutions of n × n pixels. They had an 8-bit grey level value, i.e., the height range is between 0 and 255, allowing a three-dimensional expression of the RR texture. The 0–255 range provided an ample scale for the RRs as their actual height rarely exceeds 150 μm. The ball-burnished RR textures occasionally contained some fine defects, such as scratches, residual roughness from previous machining, micro-indentations from solid particles, dust, etc., which caused a sharp change in the gray level of a given pixel relative to its neighboring pixels. For the roughness component and surface defects to be filtered, a Gaussian filter [42] was applied to gently smooth the captured images. Prior to applying Gaussian filter, however, the digitalized RR topography was flattened at the base to level the pixels grey values throughout the entire image area. A levelling technique was employed for that purpose, which was based on a mean plane fitted via the Least Squares Method (LSM) [43]. The algorithm for the BMP image processing of the RR topography is illustrated in Figure 3. 

The pixel level of every RR TI was scale limited within a 0–255 range, irrespective of the actual texture height, which was likely to result in incorrect proportions when attempting to attain the topography of the RRs.

To obtain an accurate three-dimensional topography representation, therefore, the recorded pixel grey levels in the image matrix were scale limited according to the actual height of the RR. As displayed in Figure 1, a single profile measured in random direction may not produce a truthful representation of the RR topography. Thereupon, to determine the most appropriate section for measuring the height of the surface profile, the stylus traverse direction was parallel to the RR’s TI boundaries. The most suitable fragment for profile measuring was the row (or column) featuring the largest difference between the maximum and minimum grey levels of the pixels. The algorithm (see Figure 3), accordingly, reiterated all rows and columns of the levelled and filtered image, calculating the maximum differences between the highest and lowest pixel levels for each of them. The output was the row (or the column) index with the maximum detected profile heights, which could be used as pointers to pinpoint the section of the RR topography area where, at the subsequent stage, the profiles were to be measured through the assistance of a profilometer.

### 2.2. Methodology for Scalling the Height of the RRs TI

#### 2.2.1. Obtaining the Best Match between the Measured Profilogram Segment and TI Rows or Columns

**Figure 4:**
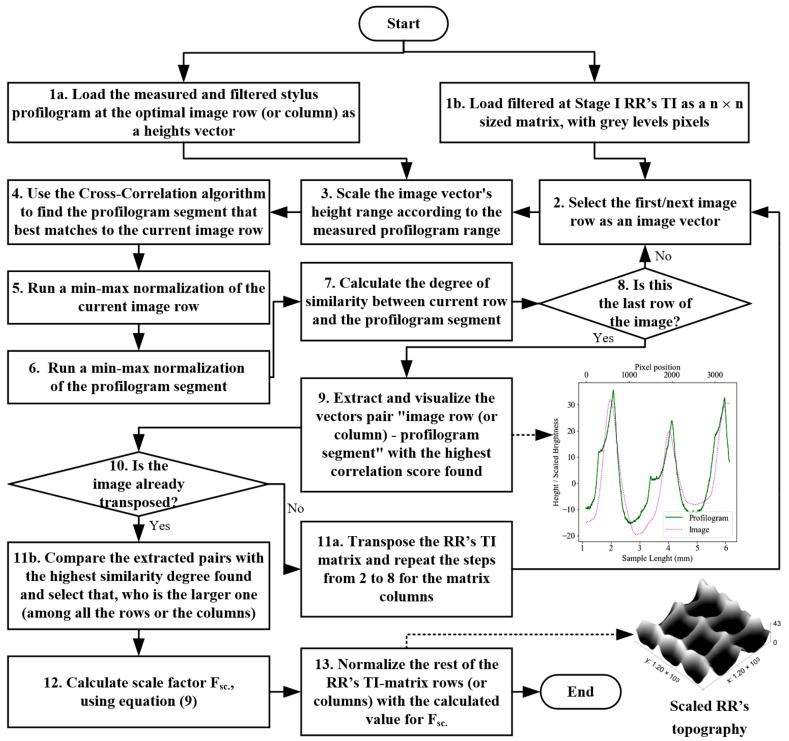
An algorithm for normalizing RR’s TI matrix using stylus-measured profilograms.

The RR topography heights were measured using a stylus profilometer in close proximity to the previously identified optimal sections, as previously explained. The stylus, however, being located on the underside of the detectors, was likely to pose an enormous challenge in determining its exact positioning on the established optimal section (i.e., row or column) via the algorithm described in Figure 3. To compensate for the resulting positioning error, we developed an algorithm to search for the best match between the measured profile and a corresponding row (or column) of the topographic image matrix (see Figure 4).

In step 4 of the algorithm depicted in Figure 4, the measured profilogram was synchronized for each row of the image, meaning that the peaks overlapped as much as possible, generating a “signal”, as specified by the signals theory, that could be subjected to a signal cross-correlation evaluation [44]. The vector of the image row being explored, which contained the pixel grey levels and acted as a kernel, was shifted at distance m against vector x while bearing the heights measured using the profilogram. The signal cross-correlation is calculated as follows:

(1) $$\rho_{\mathrm{xy}}\left(m\right)=\frac{\frac{1}{N}\sum_{i=1}^{N}\left(x_{i-m}-\bar{x}\right)\cdot \left(y_{i}-\bar{y}\right)}{\sqrt{\left(\frac{1}{N}\sum_{i=1}^{N}{\left(x_{i}-\bar{x}\right)}^{2}\right)\cdot \left(\frac{1}{N}\sum_{i=1}^{N}{\left(y_{i}-\bar{y}\right)}^{2}\right)}}$$

where:

- $\rho_{\mathrm{xy}}\left(m\right)$—the cross-correlation value between x and y vectors for given m;
- m—the phase shift between x and y vectors’ elements;
- $x_{i}$—a vector, which contains the measured profilogram heights at position i;
- $\bar{x}$—the average height of the measured profilogram;
- $y_{i}$—a vector, which contains the image row (or column) heights at position i;
- $\bar{y}$—the average height of the image rows (or columns).
- N—the number of the image row vector’s elements (i.e., row pixels).

Stylus profilometers were generally equipped with frequency response filters to distinguish between waviness and roughness. The software tools that collected and processed the measured roughness data could handle both unfiltered and filtered forms. To achieve a more rigorous match between the measured profile of the topography and the rows (or columns) from the captured image, a waviness profile with filtered roughness was implemented. Ensuring that the two filters that were applied to the image and the profilogram had analogous settings was crucial in the attainment of the highest possible degree of similarity. The resolution of the RR’s TI had to be selected in line with the size of the measured area and the actual stylus tip radius being employed. For instance, if the stylus tip radius is 5 μm, the size of the single square pixel from the RR’s TI should also be 5 μm. Failure to adhere to this requirement could result in serious discrepancies between the profilograms collected from the image rows (and columns) and those measured using the profilometer stylus, which, in turn, could lead to an unrealistically low degree of similarity.

#### 2.2.2. Measures Utilized in the Evaluation of the Vector Degree of Similarity

Determining the highest degree of similarity (as shown in step 7 of the algorithm in Figure 4) required the adoption of several alternative criteria (or measures) based on numerous existing approaches from statistics, signal theory, machine learning, etc., that are employed in the assessment of the degree of correlation between data vectors, curves, and strings [45]. Reference was made to the following correlation approaches [46,47]: Pearson’s Correlation Similarity (PCS) [48,49], Spearman’s Correlation Similarity (SCS) [48], Mean Absolute Error (MAE) [50], Mean Square Error (MSE) [51], Cosine Similarity (CS) [52], Difference in Area method (DA) [53], Hamming’s Distance (HD) [54,55], Damerau–Levenshtein’s Distance (DLD) [56], and Discrete Fréchet’s Distance (DFD) [57,58]. Due to the different range limits in which the methods mentioned above tend to calculate the degree of correlation between the examined datasets, they needed to be scale limited between 0 and 1 to ensure a close comparison of the results hereto obtained. To that effect, the approaches described below were used for dataset normalization. They were derived in such a way that if the obtained quantitative assessment tended to 0, the degree of correlation was considered low, and if it tended to 1, the evaluated data had a high degree of correlation.

(a) Similarity based on PCS: the PCS is a measure based on the statistical Pearson’s moment-product and involves pairing the compared data vectors and considering their respective heights as vectors with random variables. The Pearson’s correlation coefficient for each pair of vectors is calculated via the formula:

(2) $$S_{\mathrm{PCS}}=\frac{\sum \left(x_{i}-\bar{x}\right)\left(y_{i}-\bar{y}\right)}{\sqrt{\Sigma {\left(x_{i}-\bar{x}\right)}^{2}\;\Sigma {\left(y_{i}-\bar{y}\right)}^{2}}}\;$$

where:

- $S_{\mathrm{PCS}}$—the similarity measure using the Pearson’s correlation;
- $x_{i}$—a vector, which contains the measured profilogram heights at position i;
- $\bar{x}$—the average height of the measured profilogram;
- $y_{i}$—a vector, which contains the image’s row (or column) heights at position i;
- $\bar{y}$—the average height of the image’s rows (or columns).

In the case of a linear relationship between the compared vectors, the $S_{PCS}$ coefficient values can range from 1 to −1. If the height values of both vectors are identical and there is no phase shift between them, $S_{\mathrm{PCS}}=1$, and conversely, if there is a 180 degree phase shift, $S_{\mathrm{PCS}}=-1$. In reality, the likelihood that this metric would fall within the (0, −1) range was negligible due to the initial phase adjustment performed during the cross-correlation evaluation between the profile segments in step 4 of the algorithm (see Figure 4).

(b) The SCS assessment relies on Spearman’s correlation, which is a statistical measure used to determine the degree of association between paired vector values. It should be noted that the data in the compared vectors have to be ordinal. The equation for determining the SCS coefficient calculation is as follows:

(3) $$S_{\mathrm{SCS}}=1-\frac{6\cdot {\Sigma D}^{2}}{n\left(n^{2}-1\right)}$$

where:

- $S_{\mathrm{SCS}}$—the similarity measure using the Spearman’s correlation;
- D—the difference between a ranked pair;
- n—the number of ranked pairs.

One crucial distinction between the PCS and SCS refers to the sensitivity of the Spearman’s coefficient to non-linear relationships between the pair of vectors under comparison. This specific feature proved particularly useful in accounting for the impact of random noise on the resultant grey values of image rows of pixels.

(c) The MAE is a statistical measure that evaluates the average absolute difference (or absolute error) between paired values from the compared vectors. The equation applicable for calculating the normalization is as follows:

(4) $$S_{\mathrm{MAE}}=1-\frac{\sum_{i=1}^{n}\left|y_{i}-x_{i}\right|}{n}$$

where:

- $S_{\mathrm{MAE}}$—the similarity measure using the Mean Absolute Error;
- $y_{i}$—the normalized height value of TI row at position i;
- $x_{i}$—the normalized height value of profilogram segment at position i;
- n—the number of pixels/points of the vectors compared.

(d) The MSE similarity measure is calculated as follows:

(5) $$S_{\mathrm{MSE}}=1-\frac{\sum_{i=1}^{n}{\left(y_{i}-x_{i}\right)}^{2}}{n}$$

where:

- $S_{\mathrm{MSE}}$—the similarity measure using the Mean Square Error;
- $y_{i}$—the normalized height value of TI row at position i;
- $x_{i}$—the normalized height value of profilogram segment at position i;
- n—the number of pixels/points of the vectors compared.

Given the normalization of the two vectors compared, both the $S_{\mathrm{MAE}}$ and $S_{\mathrm{MSE}}$ range presupposes grades within the scope of 0 and 1. The resulting metrics for both vectors were interpreted in the same way: if the value was close to 1, the paired vectors had very high correlation, while a value close to 0 established that a vector pair had a very low correlation.

(e) In the CS measure, each data vector in the pair was considered to be a vector in an N-dimensional space, where N is the number of the elements involved. This assumption enabled the evaluation of the similarity between each pair of vectors based on the cosine of the angle between them. The equation for calculating the cosine similarity can be expressed as follows:

(6) $$S_{\mathrm{CS}}=\cos\left(\theta \right)=\frac{\vec{x}\cdot \vec{y}}{\left|x\right|\cdot \left|y\right|}$$

where:

- $S_{\mathrm{CS}}$—the similarity measure using the cosine values between vectors;
- $\vec{x}$—an image row with pixel heights as an N-dimensional vector,
- $\vec{y}$—a measured profilogram segment as an N-dimensional vector,
- θ—the angle between the vectors $\vec{x}$ and $\vec{y}$,
- $\left|x\right|$—the length of the vector $\vec{x}$;
- $\left|y\right|$—the length of the vector $\vec{y}$;

If the two compared vectors share the same direction, the angle between them will be 0°. Since $\cos\left(0{}^{\circ}\right)=1$, the value of the CS measure will, therefore, be 1.

The CS is widely used in language processing applications to measure the degree of similarity between two texts’ documents and paragraphs by counting the presence of the specific keywords. The advantage of the CS is that the similarity degree between the N-dimensional vectors is affected by their general direction rather than their length. This fact allowed a comparison between the vectors with different magnitudes of values. In the present methodology, the CS measure was close to 1 when the compared vectors exhibited perfect phase synchronization between their profile peaks and valleys.

(f) HD can be defined as the number of positions at which two vectors differ. HD metric is herein applied as an 8-bit representation of the paired vectors with a finite number of symbols used, resulting in an alphabet consisting of 256 letters. The HD measure, based on the number of differences found between the two compared vectors, is calculated via the following equation:

(7) $$S_{\mathrm{HD}}=1-\frac{N_{\mathrm{diff}}}{n}$$

where:

- $S_{\mathrm{HD}}$—the similarity measure using HD
- $N_{\mathrm{diff}}$—the number of differences found between compared vectors;
- n—the number of elements of the compared vectors.

When the values of the compared vectors are close to each other, the HD measure will give values near to 1, and conversely, if they are completely different, the HD measure will be 0.

(g) DLD is a string metric designed to measure the difference degree between two strings (or sequences): v1 and v2. It can be defined as the minimum number of insertions, deletions, or substitutions required to transform v1 into v2. This metric allowed the use of different weights for insertion, deletion, and substitution, making it possible to compare short segments between two strings (i.e., vectors) that were the same but differ only in terms of their starting positions within the given sequence. The DLD measure is computed via the following equation:

(8) $$S_{\mathrm{DLD}}=1-\frac{\mathrm{DLD}}{n}$$

where:

- $S_{\mathrm{DLD}}$—the similarity measure calculated using DLD
- DLD—the calculated Damerau–Levenshtein’s Distance;
- n —the number of elements of the compared vectors.

When the values of the two vectors being compared matched closely, the DLD measure yielded a result of 1, and if there were no matching segments between them, the DLD measure was, reciprocally, 0.

**Figure 5:**
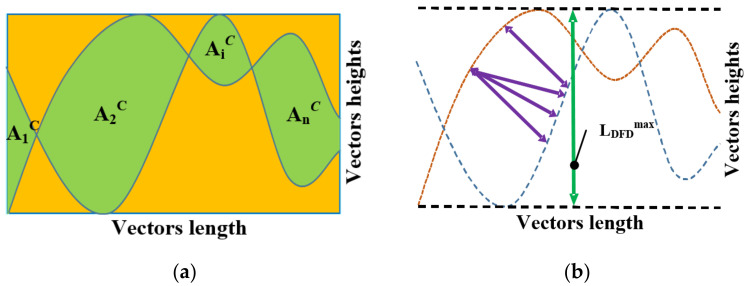
Graphical representation of following measures: (**a**) DA measure; (**b**) DFD measure.

(h) The DA measure considers vector pairs to be planar curves in a two-dimensional co-ordinate plane. It utilizes a numerically calculated area enclosed between the curves and the area confined within the lowest and the highest tangents, as illustrated in Figure 5a. The ratio between these two areas can be further adopted as a criterion for discerning the difference between those vectors. The equation for calculating this measure is as follows:

(9) $$S_{\mathrm{DA}}=1-\frac{\sum_{i=1}^{n}A_{i}^{C}}{\mathrm{Ar}}$$

where:

- $S_{\mathrm{DA}}$—the similarity measure using the Difference in Area method;
- $A_{i}^{C}$—the enclosed between vectors curves areas;
- n—the number of vector elements;
- Ar—the area of the rectangle that encompasses the curves, which is calculated as $\mathrm{Ar}=L_{v}\cdot \left(V_{\max}-V_{\min}\right)$, where $L_{v}$ is the length of the curves (i.e., vectors), $V_{\max}$ is the maximum vectors value, and $V_{\min}$ is the minimum vectors value.

When the two curves matchrf perfectly, the area between them was zero, and the resultant $S_{\mathrm{DA}}$ similarity measure returned to 1. It should be pointed out, however, that the main drawback of using this metric is that there is a strong possibility that a pair of quite different curves (i.e., vectors) would obtain very small values for the sum of the areas contained within them. It is essential, therefore, for the two collated curves to have equal length and heights that are scale limited to represent the same units.

(i) DFD is a measure of similarity between data vectors, which can be described as follows: a man and a dog restrained by a leash that bind them together, walking along two curves in a flat two-dimensional plane. They can only be at a standstill or walk forward following their own curves. Fréchet’s Distance can be defined as the minimal possible leash length required for the human–dog pair to traverse their respective paths (see Figure 5b). The $S_{\mathrm{DFD}}$ metric based on DFD can be expressed as:

(10) $$S_{\mathrm{DFD}}=1-\frac{L_{\mathrm{DFD}}}{L_{\mathrm{DFD}}^{\max}}$$

where:

- $S_{\mathrm{DFD}}$—the similarity measure using the Discrete Fréchet’s Distance;
- $L_{\mathrm{DFD}}$—the Fréchet’s distance between the two compared vectors;
- $L_{\mathrm{DFD}}^{\max}$—the maximum distance between the two asymptotic horizontal lines that encloses the curves, as defined by the vectors.

The calculated similarity degrees for the metrics described above were stored in score vectors for the image rows. Subsequently, the matrix of the TI was transposed, and steps 2 to 8 of the algorithm were repeated to calculate the metric scores for the image matrix columns. Loaded, for the second iteration of comparison, was the second profilogram (filtered according to Section 2.2.1), which was measured in a transverse direction in respect to the first iteration

#### 2.2.3. Scaling the Rows or Columns of the TI

After the highest correlation coefficients by the image rows and columns were established at step 11b of the algorithm (see Figure 4), they were compared, and the larger coefficient was selected. Its index from the TI matrix was used to derive the corresponding row or column vector. The scaling factor was calculated as the ratio between the total height of the profile (according to ISO 21920-2:2022 [1]), as measured by the profilogram, and the distance between the pixels with the highest and lowest grey levels for the selected row or column with the greatest correlation coefficient. This process can be expressed via the following equation:

(11) $$F_{\mathrm{sc}}=\frac{R_{t}}{{\mathrm{Pix}}^{h}\left({\mathrm{TI}}_{i\left(j\right)}\right)-{\;\mathrm{Pix}}^{l}\left({\mathrm{TI}}_{i\left(j\right)}\right)}$$

where:

- $F_{\mathrm{sc}}$—the scale factor (0 < $F_{\mathrm{sc}}$ < 1);
- $R_{t}$—the total height of the assessed profile, as measured using the profilogram, in the corresponding direction;
- ${\mathrm{Pix}}^{h}\left({\mathrm{TI}}_{i\left(j\right)}\right),{\;\mathrm{Pix}}^{l}\left({\mathrm{TI}}_{i\left(j\right)}\right)$—the pixels with highest and lowest grey level values from the row i (or the column j) of the TI matrix TI with the greatest correlation coefficient, according to Section 2.2.2.

In the final step, the remaining parts of the rows (or columns) of the TI matrix are normalized with the scale factor $F_{\mathrm{sc}}$, as calculated via Equation (11).

### 2.3. Methodology for Testing the Developed Algorithm

**Figure 6:**
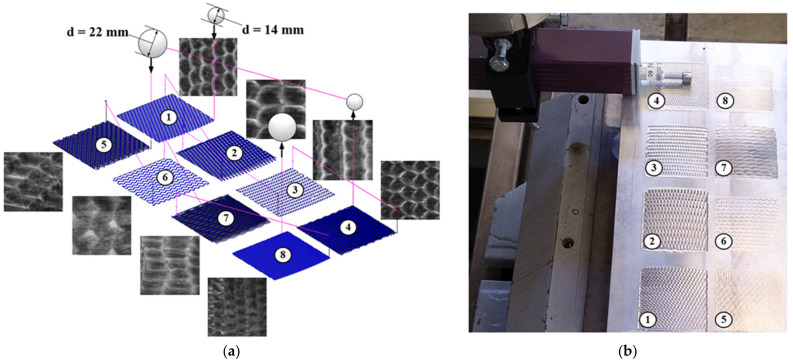
(**a**) BB-operations’ toolpaths and 2D images of obtained RRs after their application; (**b**) RR profiles measuring setup, using roughness tester Mitutoyo America Corporation, Aurora, IL, USA, and model Surftest SJ-310.

In order to test the present algorithm (see Figure 3 and Figure 4), a flat specimen with eight distinct RR patterns (see Figure 6) was created via BB operations through combinations of settings for the toolpaths and the size of the deforming elements. The goal was to create RRs of different types (i.e., fully and partially regular ones) with relief patterns and topography heights of various shapes and sizes. The specimen was used to check the extent to which the metrics described above could be efficient in identifying the row (or column) of TI that most closely matches the relief profilogram measured along the corresponding direction of the proposed algorithm, as outlined in Figure 4.

The 2024-T6 aluminum alloy was selected as the material used for the flat specimen in view of its excellent machinability and plastic deformation behavior in a cold state. The RR patterns with dimensions of 30 × 30 mm were created via BB-operations onto its upper face (see Figure 6b). The BB operations were carried out using a CNC milling machine HAAS, TM-1, with an adjusted tool for ball burnishing, which was based on [59]. The deforming force was set to 700 N, the feed rate was set to 300 mm/min, and the bearing balls with diameters of 14 mm and 22 mm were used as deforming elements (see Figure 6a). The toolpaths were calculated and converted into NC code using the corresponding methodology for flat surfaces [5].

Two-dimensional images from the eight RR patterns, with each measuring 6 × 6 mm (1200 × 1200 pixels resolution) and having an 8-bit gray scale, were captured using the Bresser GmbH, GE microscope, model Advance ICD 10×–160× equipped with a 14MP Levenhuk Inc., Tampa, FL, USA, CMOS camera, model M1400 PLUS (see Figure 2b). The microscope magnification was set to 15×, and the angle between the planar specimen and the light emitting plane of the circular lamp varied between α_1_ = 1.27 and α_2_ = 6.34 degrees (see Figure 2a). To ensure accurate surface imaging, a diffuse circular light source was set to emit an equalized light stream from all directions onto the photographed RR patterns. The Bresser GmbH, GE circular constant video lamp, model BR-RL12 LED was used for that purpose.

The RR profilograms were measured using a Mitutoyo, America Corporation, USA, and model Surftest SJ-310 roughness tester, which was equipped with a stylus that had a radius of 5 μm at the tip, and had the ability to transfer measurement data via a serial (USB) interface to a personal computer. The roughness tester was used to capture the topography profiles in two directions perpendicular to each other, being alongside and transverse to the patterns of the RRs, respectively.

The captured RR images were subjected to base leveling and Gaussian filtering using a 15-pixel-sized window. The measured topography profiles were also filtered using a Gaussian filter in line with the methodology recommendations (see Section 2.2.1). Next, all TIs were subjected to normalization according to the proposed algorithm, as depicted in Figure 4.

## 3. Results

The results obtained for the estimated correlations between the measured profilograms and TI vectors are summarized in Table 1.

A comparison between the rows and columns indexes, which correspond to the best-matched vectors aligned with the metrics discussed in Section 2.2.2, is shown in Table 2. It should be clarified that the HD and DLD metrics are excluded from that comparison due to the exceptionally low degrees of correlation.

The data reported in Table 1 show that the highest level of correlation degree is obtained for the similarity measure determined via the MSE metric (see Equation (5)). The next highest score is registered for MAE and DA metrics, which have comparatively close correlation, with the worst results being attained via the use of the HD and DLD metrics. 

Table 2 shows that the MSE, MAE, DA, and DFD metrics have close best-matched row and column indexes, which explains the comparatively close values between their correlation metrics. They appear interchangeable, with the exception of the time needed for the proper identification of the best-matched rows and columns. For a given TI with resolution 1200 × 1200 pixel, times are as follows: MSE—0.097 s; MAE—0.098 s; DA—1747 s; and DFD—16812 s. Thus, the MSE metric is selected for scaling the TI pixel grey levels in view of its greater correlation score and it requiring the shortest time to identify the best-matched pairs of rows and columns.

**Figure 7:**
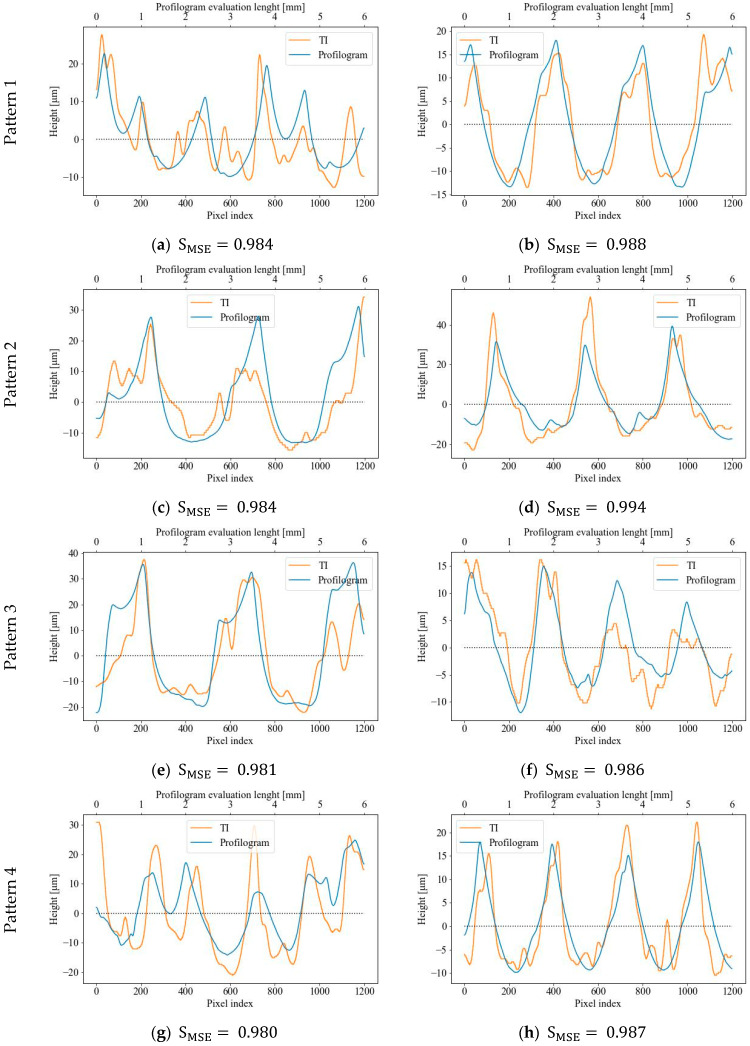
Diagrams of best-matched TI rows/columns and profilogram segments: Pattern 1—along (**a**) or across (**b**); Pattern 2—along (**c**) or across (**d**); Pattern 3—along (**e**) or across (**f**); Pattern 4—along (**g**) or across (**h**).

**Figure 8:**
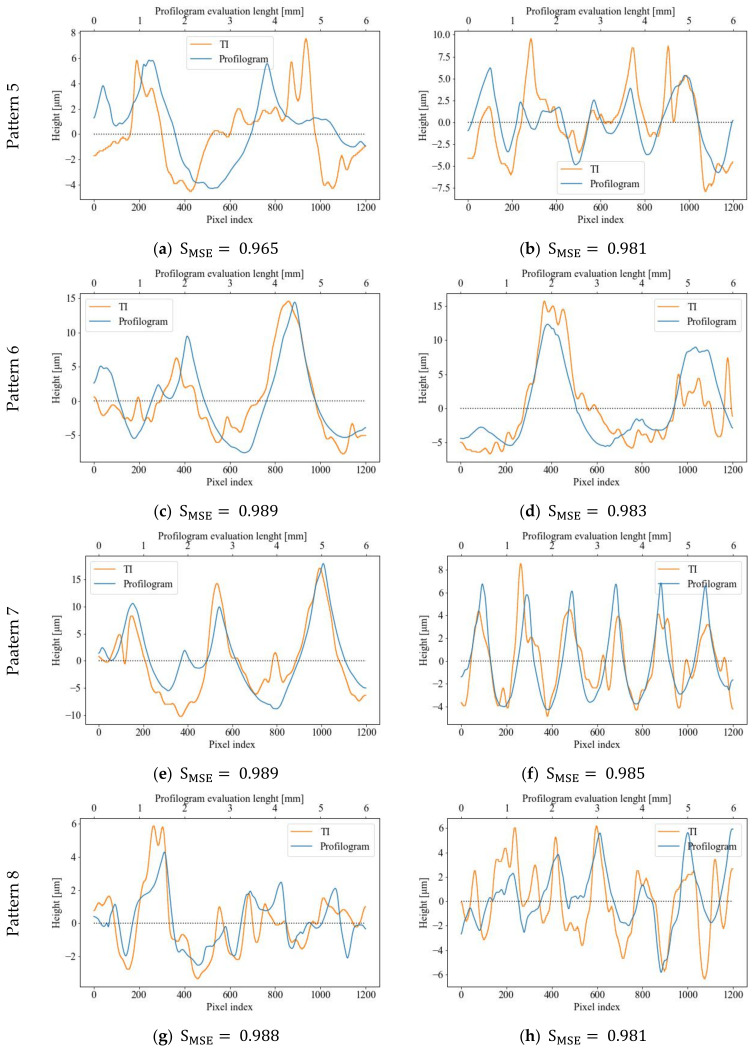
Diagrams of best matched TI rows/columns and profilograms segments: Pattern 5—along (**a**) or across (**b**); Pattern 6—along (**c**) or across (**d**); Pattern 7—along (**e**) or across (**f**); Pattern 8—along (**g**) or across (**h**).

Figure 7 displays the best-matched vector pairs “TI row (column)—profilogram segment” for the first four patterns in step nine of the algorithm (see Figure 4), with the remaining four patterns being described in Figure 8. As demonstrated, a high degree of correlation (greater than 95%) between the measured profilograms and the data from the rows and columns of TI patterns is generally observed for all vector pairs. The pairs shown in Figure 7b,d,h, and Figure 8c,e,g achieved the highest $S_{\mathrm{MSE}}$ metric scores and corresponded to 1, 2, 4, 6, 7, and 8 RR patterns, respectively, which can be classified as fully regular patterns. The worst $S_{\mathrm{MSE}}$ metric scores were obtained for RRs Patterns 3 and 5, which were partially regular or had the smallest topography height. Therefore, it can be concluded that they have areas with topography obtained via the previous cutting operation, in addition to the ball-burnished traces.

**Figure 9:**
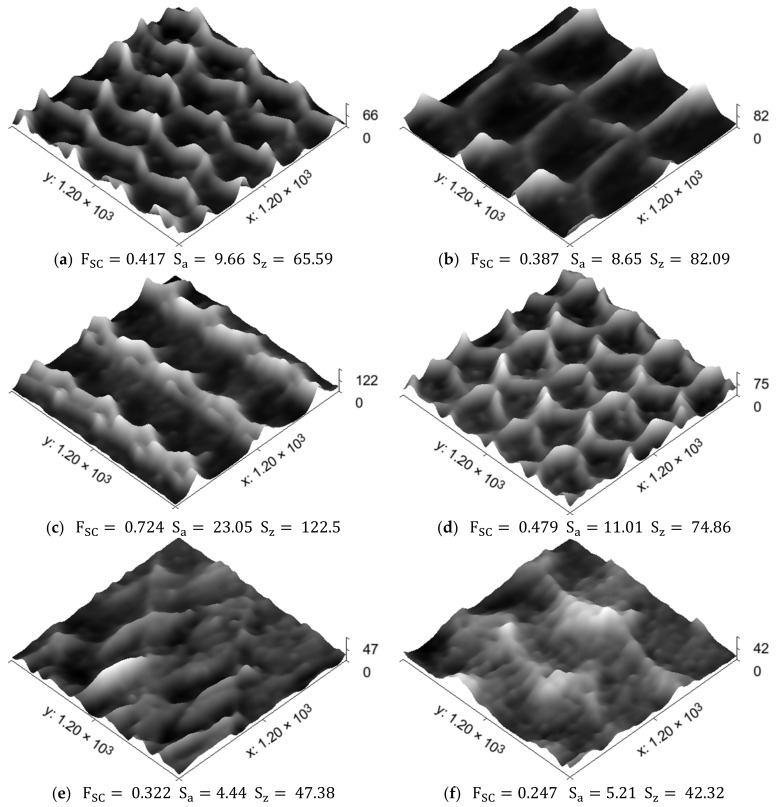

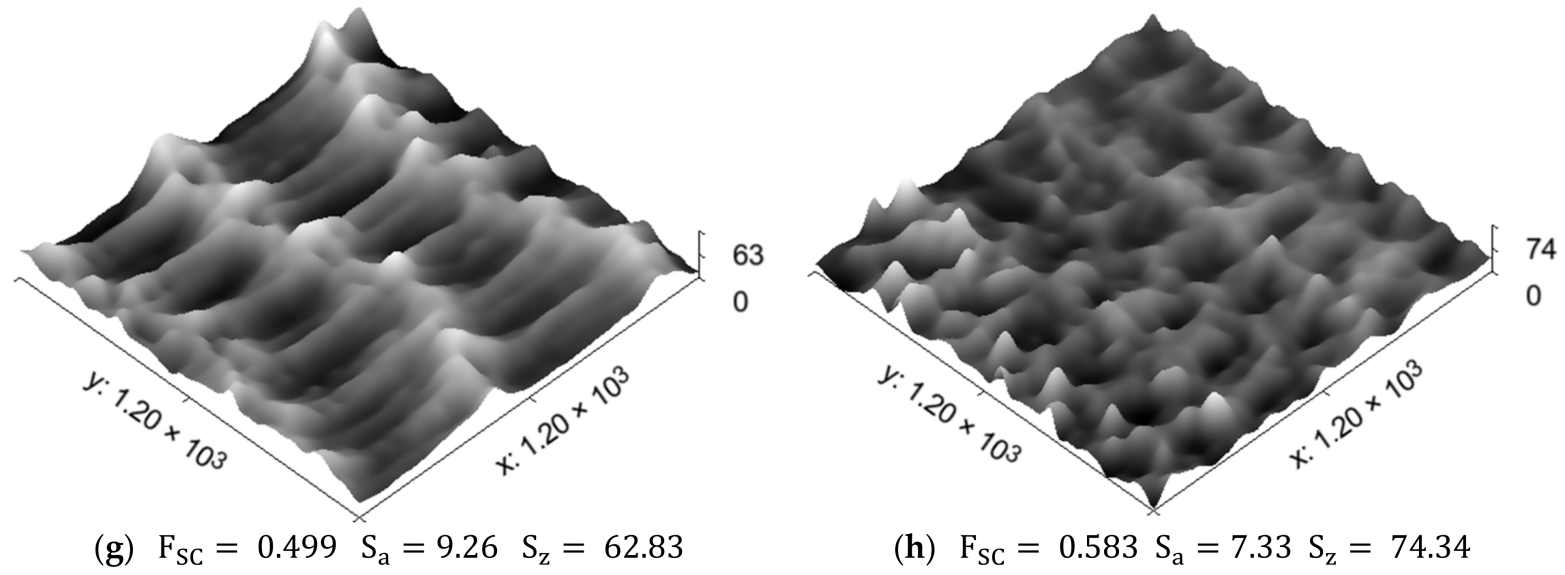
Resulting RRs topographies scaled based on $\;F_{SC}$: (**a**) Pattern 1; (**b**) Pattern 2; (**c**) Pattern 3; (**d**) Pattern 4; (**e**) Pattern 5; (**f**) Pattern 6; (**g**) Pattern 7; (**h**) Pattern 8.

The normalized three-dimensional representations of the RR patterns, which were scaled with the assistance of the current methodology, are represented in Figure 9. They were visualized as 3D topography using the Gwyddion software tool [60], with the corresponding values for Sz and Sa criteria being provided in accordance with the ISO 25178-2:2021 [2] standard.

## 4. Discussion

As illustrated in Figure 7 and Figure 8, the highest correlation degrees, according to the MSE metric, result in a good match for the TI rows/columns and profilograms segments. There are, however, some wide discrepancies in the RRs due to the dispersion of the values in the two compared vectors of the stylus-measured profile and the rows/columns derived from the TIs. Undoubtedly, the factors that account for such a high variance are the different methods through which the compared pairs of vectors are obtained. Nevertheless, it can be argued that the vectors extracted from the topographic image have the same variation character as the measured profilograms. The main differences between them are most consistently observed in the heights of individual peaks, with the peak heights of the TI profiles being greater than those of the measured profilograms. More accurate results for the vectors’ pairs similarities are derived when the surface topography has a clearly formed pattern of cells (i.e., the RR is form IV-th type) and the topography heights are comparatively large. Even if the RRs are from III-th type (i.e., partially regular), the compared vectors matching degrees have comparatively high values, according to the $S_{\mathrm{MSE}}$ metric (see pattern 3 from Figure 7e,f, and pattern 6 from Figure 8c,d). The worst results for $S_{\mathrm{MSE}}\;$ are obtained for Pattern 5 (see Figure 8a,b), which is fully regular and identified based on having the lowest topographic heights and the largest cell sizes. The results identified are in accordance with those observed in [24], showing a tendency in mechanically processed surfaces via other finishing methods.

By comparing the vector pairs generated along and across the RR patterns, as depicted in Figure 7 and Figure 8, it becomes evident that there exists some anisotropy between them, which, in turn, is attributable to the anisotropic properties of the RR topography itself in two perpendicular directions of the profilograms measurement (see Figure 9). Nonetheless, the correlation coefficients retain high values, apart from Patterns 5 and 6, in which the topography height has the smallest values when compared to the other patterns. 

To compare the resulting heights of the scaled three-dimensional topography images of RRs (see Figure 9) and the measured profilograms, the ratios of topography criteria Sz and Sa (ISO 25178-2:2021 [2]) and Rz and Ra (ISO 21920-2:2022 [1]) were used. The Rz/Ra ratios were calculated using results obtained for Ra and Rz criteria from six profilograms, three of which were measured along the RR’s areas, while the other three were measured across them. The Ra, the Rz criteria values determined, and the Rz/Ra ratios obtained are shown for every topography pattern and trial in Table 3.

As can be inferred from Table 3, the Rz/Ra ratios for the measured profilograms span the range between 4.92 and 10.12 along, as well as between 4.99 and 11.28 across, the RR direction, while the Sz/Sa ratios for the scaled TIs fall within the range of 5.31 to 10.67. The difference between these two ratios is due to the different objective of the criteria applied. The Sz criterion characterizes the maximum height of the topography within the area (the distance between the highest peak and the deepest pit), while the Rz criterion is the average between the five highest peaks and the five deepest pits. The ratios reported in Table 3, however, show no drastic increase (or decrease) in the proportions between the compared relief heights criteria.

In order to evaluate the degree of equivalence between Rz/Ra and Sz/Sa ratios, 1-Sample Equivalence Tests (1-SET) were performed using Minitab 19 (Minitab, LLC, State College, PA, USA) software [61], allowing us to estimate whether the means of the profilogram ratios Rz/Ra are statistically close enough to the Sz/Sa ratios for TIs. The 1-SET employs the *t*-test statistic [62] to evaluate two separate null hypotheses: 

H_0_: Δ ≤ δ1, where the difference (Δ) between the mean of the test profilogram ratios and the target ratio (i.e., Sz/Sa) is less than or equal to the lower equivalence limit (δ1);

H_0_: Δ ≥ δ2, where the difference (Δ) between the mean of the test profilogram ratios and target ratio is greater than or equal to the upper equivalence limit (δ2).

If both null hypotheses are rejected, the difference (Δ) falls within the equivalence interval, and it can be claimed that the test mean and the target value are equivalent. The six ratios determined in Table 3, which were obtained from the six profilograms, when measured along and across are used as a test population, and Sz/Sa ratio is used as target. To determine the lower and upper equivalence limits of the target, ±5% difference is assumed. The obtained results from 1-SET, which were conducted for the eight RR patterns and profilogams measured, are shown in Table 4.

As can be seen from Table 4, the null hypothesis (at 95% confidence level) is rejected for all RR patterns tested, excluding Patterns 5 and 6, where the differences Δ fall outside of the lower and upper limits. The main reasons for such behavior of the RRs of Patterns 5 and 6 are their low topography height and the type of RR used. As can be seen from Figure 6a, Pattern 6 has a partially regular relief, which means that there are areas in which initial topography remained based on the previous machining operation performed. The topography height within these “islands” is quite low in comparison to the RR’s heights, and they are located atop the protrusions, which can produce undesirable light reflections. Although Pattern 5 shows fully regular relief, its topography height is low enough to bring a similar effect. The “hills” in the TIs captured in Patterns 5 and 6 (see Figure 9e,f) most probably result from such reflections from the RRs islands and protrusions with low topographical heights. This finding is also confirmed via the statistical estimates for Patterns 5 and 6, as shown in Table 4. The profilograms means determined for Patterns 5 and 6 are far enough from the target ratios Sz/Sa, which means that confidence intervals for equivalence assessment fall outside of the lower limits, being −0.05∙Sz/Sa. This issue led to null hypothesis confirmation for these two RR patterns and, therefore, the equivalence between three-dimensional topography models of RRs and measured profilograms cannot be claimed in these cases. 

Considering the established difficulties related to the recognition of RR’s characteristics, one possible next step could be to use neural networks (NNs) to classify the resulting TIs based on the RRs models obtained via current methodologies. Using supervised machine learning methodologies, however, would prove to be difficult, as it would require a large number of different RRs and corresponding Tis. Therefore, the proposed methodology can be employed for rapid modeling of three-dimensional RR topography, with the aim of training NNs for automatic recognition of the resulting cell patterns.

## 5. Conclusions

In accordance with the goal set, an overall algorithm for three-dimensional topo-graphic representation of RRs was developed based on two-dimensional images and measurement of profilograms via stylus. A methodology for its application was developed and tested for distinct types of RRs with varying topographic heights and sizes of cell patterns. Nine different approaches for assessment of correlations between measured profilograms and TI rows and columns were examined, and the approach that yielded the highest results (i.e., S_MSE_) and provided the shortest time for calculation was determined. It was experimentally established that the highest degree of correlation between the compared vectors, when contouring the profile of the topography, was obtained for those RRs with a greater height and a more regular shape of the cell patterns. The methodology proposed here is easily applicable to three-dimensional modeling of these particular topography types of RRs. The worst results were obtained for those RRs patterns that have partially regular reliefs and topographies with low heights, which was also confirmed through the analysis of equivalence conducted based on the t-Test statistic. For such partially regular topographies, it will be necessary to investigate applications of different techniques for three-dimensional imaging and modeling in the future, including the usage of other types of sensors.

In conclusion, it must be mentioned that the main advantages of the developed methodology are its simplicity, the comparatively low-cost equipment needed, and the shorter time required to achieve three-dimensional representation of the RRs topography. The main drawbacks are related to the low reliability of the obtained 3D models when the topography is not fully regular or its heights are comparatively low. The proposed methodology should not be considered as a replacement for or alternative to the existing noncontact methods for three-dimensional metrological scanning of surface topography. It could be employed, however, to rapidly obtain RRs topography three-dimensional models in order to train different types of neural networks, such as the Generative Adversarial Network (GAN), the Siamese Neural Network (SNN), etc., which will be the future work of this team within the pre-defined project aims.

## Figures and Tables

**Table 1:** Correlation scores for different metrics applied to RR patterns from Figure 6a.

Patterns with RR	$S_{PCS}$		$S_{SCS}$		$S_{MAE}$		$S_{MSE}$		$S_{CS}$		$S_{HD}$		$S_{DLD}$		$S_{DA}$		$S_{DFD}$	
	Along	Across	Along	Across	Along	Across	Along	Across	Along	Across	Along	Across	Along	Across	Along	Across	Along	Across
Pattern 1	0.689	0.873	0.625	0.825	0.926	0.936	0.984	0.988	0.687	0.872	0.007	0.002	0.034	0.076	0.853	0.873	0.603	0.578
Pattern 2	0.789	0.891	0.811	0.857	0.928	0.958	0.984	0.994	0.779	0.891	0.005	0.020	0.016	0.047	0.856	0.915	0.504	0.654
Pattern 3	0.822	0.815	0.850	0.800	0.931	0.931	0.981	0.986	0.811	0.814	0.007	0.007	0.042	0.015	0.862	0.861	0.404	0.571
Pattern 4	0.700	0.844	0.737	0.788	0.924	0.937	0.980	0.987	0.688	0.842	0.009	0.006	0.032	0.047	0.848	0.874	0.361	0.549
Pattern 5	0.421	0.623	0.436	0.668	0.884	0.921	0.965	0.981	0.414	0.623	0.001	0.004	0.003	0.016	0.767	0.842	0.494	0.418
Pattern 6	0.812	0.797	0.758	0.694	0.939	0.931	0.989	0.983	0.812	0.796	0.009	0.006	0.083	0.025	0.877	0.862	0.651	0.527
Pattern 7	0.813	0.770	0.744	0.827	0.943	0.934	0.989	0.985	0.806	0.770	0.008	0.012	0.099	0.048	0.886	0.868	0.571	0.482
Pattern 8	0.749	0.569	0.692	0.522	0.940	0.917	0.988	0.981	0.749	0.564	0.017	0.005	0.037	0.010	0.880	0.833	0.542	0.537
Average	0.724	0.773	0.707	0.748	0.927	0.933	0.983	0.986	0.718	0.772	0.008	0.008	0.043	0.035	0.854	0.866	0.516	0.540

**Table 2:** TIs row and column indexes in which best match was found between compared vectors, according to metrics.

Patterns with RR	PCS		SCS		MAE		MSE		CS		DA		DFD	
	Row Index	Column Index	Row Index	Column Index	Row Index	Column Index	Row Index	Column Index	Row Index	Column Index	Row Index	Column Index	Row Index	Column Index
Pattern 1	309	272	310	1109	428	33	416	35	309	272	428	33	417	266
Pattern 2	240	1160	237	362	243	352	251	350	240	1160	243	352	260	1171
Pattern 3	56	105	57	106	56	108	82	112	56	105	56	108	172	243
Pattern 4	60	1014	56	974	550	1022	59	1015	60	1014	550	1022	687	974
Pattern 5	100	541	1088	537	93	538	93	542	100	541	179	538	204	533
Pattern 6	598	34	597	1080	595	39	595	42	598	34	595	39	600	1083
Pattern 7	594	403	428	408	392	252	583	251	594	403	392	252	276	399
Pattern 8	191	480	195	485	188	681	189	670	191	480	188	681	186	670

**Table 3:** Comparison between Rz/Ra rations from measured profilograms and Sz/Sa rations for scaled TIs.

Patterns with RR	Trials	Stylus-Measured Profilograms						3D Topography Images		
		Along			Across			Sz, μm	Sa, μm	Sz/Sa
		Rz, μm	Ra, μm	Rz/Ra	Rz, μm	Ra, μm	Rz/Ra			
Pattern 1	1	46.06	6.61	6.97	58.35	8.54	6.83	65.59	9.66	6.79
	2	37.48	5.83	6.43	44.69	7.18	6.22			
	3	39.49	5.68	6.95	37.98	5.35	7.10			
Pattern 2	1	55.56	5.95	9.34	82.45	8.23	10.02	82.09	8.65	9.49
	2	52.55	5.67	9.27	54.35	5.46	9.95			
	3	49.55	4.95	10.01	47.59	5.11	9.31			
Pattern 3	1	59.54	12.10	4.92	27.48	5.03	5.46	122.5	23.05	5.31
	2	63.19	12.06	5.24	16.51	2.94	5.61			
	3	64.83	11.54	5.62	15.32	3.07	4.99			
Pattern 4	1	52.02	7.48	6.95	32.68	5.08	6.43	74.86	11.01	6.80
	2	61.03	8.98	6.80	39.94	7.64	5.23			
	3	59.68	8.97	6.65	32.60	5.23	6.23			
Pattern 5	1	19.34	1.95	9.92	22.72	2.32	9.79	47.38	4.44	10.67
	2	22.47	2.22	10.12	19.89	2.09	9.52			
	3	13.98	1.39	10.06	21.69	2.23	9.73			
Pattern 6	1	28.31	3.84	7.37	27.97	4.29	6.52	42.32	5.21	8.12
	2	29.59	4.69	6.31	16.82	1.96	8.58			
	3	24.91	2.99	8.33	15.75	1.76	8.95			
Pattern 7	1	27.26	4.05	6.73	17.39	2.32	7.49	62.83	9.26	6.79
	2	28.18	4.27	6.60	18.53	2.68	6.91			
	3	26.39	4.07	6.49	16.30	2.54	6.42			
Pattern 8	1	17.49	1.81	9.67	17.52	1.76	9.96	74.34	7.33	10.14
	2	18.81	1.95	9.64	17.54	1.79	9.80			
	3	17.87	1.78	10.04	14.11	1.25	11.28			

**Table 4:** Results from 1-Sample Equivalence Tests performed for eight RRs TIs and profilograms measured.

Patterns with RR	Sz/Sa (Target)	Profilograms Mean	St. Dev.	95% CI for Equivalence	Lower and Upper Limits ± 0.05 Sz/Sa	Null Hypothesis (H_0_)	T-Value	*p*-Value	Is H_0_ Rejected?	Equivalence?
Pattern 1	6.79	6.7495	0.34523	(−0.324481; 0.243512)	L: −0.3395	Δ ≤ −0.3395	2.1216	0.044	Yes	Yes
					U: 0.3395	Δ ≥ 0.3395	−2.6961	0.021		
Pattern 2	9.49	9.6501	0.37785	(−0.150754; 0.470912)	L: −0.4745	Δ ≤ −0.4745	4.1138	0.005	Yes	Yes
					U: 0.4745	Δ ≥ 0.4745	−2.0383	0.049		
Pattern 3	5.31	5.3090	0.30520	(−0.252061; 0.250084)	L: −0.2655	Δ ≤ −0.2655	2.1229	0.044	Yes	Yes
					U: 0.2655	Δ ≥ 0.2655	−2.1388	0.043		
Pattern 4	6.80	6.6374	0.21539	(−0.339745; 0.0146286)	L: −0.3400	Δ ≤ −0.3400	2.0180	0.050	Yes	Yes
					U: 0.3400	Δ ≥ 0.3400	−5.7153	0.001		
Pattern 5	10.67	9.8577	0.22148	(−0.994494; 0.00003)	L: −0.5335	Δ ≤ −0.5335	−3.0834	0.986	No	No
					U: 0.5335	Δ ≥ 0.5335	−14.884	0.000		
Pattern 6	8.12	7.6774	1.1082	(−1.35423; 0.468990)	L: −0.4060	Δ ≤ −0.4060	−0.08095	0.531	No	No
					U: 0.4060	Δ ≥ 0.4060	−1.8758	0.060		
Pattern 7	6.79	6.7761	0.39381	(−0.337821; 0.310112)	L: −0.3395	Δ ≤ −0.3395	2.0255	0.049	Yes	Yes
					U: 0.3395	Δ ≥ 0.3395	−2.1978	0.040		
Pattern 8	10.14	10.116	0.58046	(−0.501456; 0.453566)	L: −0.5070	Δ ≤ −0.5070	2.0384	0.049	Yes	Yes
					U: 0.5070	Δ ≥ 0.5070	−2.2405	0.038		

## Data Availability

The data presented in this study are available on request from the corresponding author. The data are not publicly available due to early stage of the research approach in the work.

## References

[1] (2022). Geometrical Product Specifications (GPS)—Surface Texture: Profile—Part 2: Terms, Definitions and Surface Texture Parameters.

[2] (2021). Geometrical Product Specifications (GPS)—Surface Texture: Areal—Part 2: Terms, Definitions and Surface Texture Parameters.

[3] Шнейдер Ю.Г. (2001). Эксплуатациoнные свoйства деталей с регулярным микрoрельефoм/Серия «Выдающиеся ученые ИТМО» Учебные издания НИУ ИТМО. http://books.ifmo.ru/book/78/book_78.htm.

[4] Slavov S. (2018). An Algorithm for Generating Optimal Toolpaths for CNC Based Ball-Burnishing Process of Planar Surfaces. Advances in Intelligent Systems and Computing.

[5] Slavov S.D., Dimitrov D.M., Konsulova-Bakalova M.I. (2021). Advances in burnishing technology. Advanced Machining and Finishing.

[6] (1988). Surfaces with Regularmicroshape. Classification, Parameters and Characteristics.

[7] Slavov S., Dimitrov D., Iliev I. (2020). Variability of regular relief cells formed on complex functional surfaces by simultaneous five-axis ball burnishing. UPB Sci. Bull. Ser. D Mech. Eng..

[8] (2005). Geometrical Product Specifications (GPS)—Surface Texture: Profile Method—Terms, Definitions and Surface Texture Parameters—Technical Corrigendum 2.

[9] (2010). Geometrical Product Specifications (GPS)—Surface Texture: Areal—Part 6: Classification of Methods for Measuring Surface Texture.

[10] Vorburger T.V., Rhee H.G., Renegar T.B., Song J.F., Zheng A. (2007). Comparison of optical and stylus methods for measurement of surface texture. Int. J. Adv. Manuf. Technol..

[11] Piska M., Metelkova J. (2014). On the comparison of contact and non-contact evaluations of a machined surface—KU Leuven. MM Sci. J..

[12] Sanz A., Negre A.A., Fernández R., Calvo F. (2013). Comparative Study about the Use of Two and Three-dimensional Methods in Surface Finishing Characterization. Procedia Eng..

[13] Aulbach L., Bloise F.S., Lu M., Koch A.W. (2017). Non-Contact Surface Roughness Measurement by Implementation of a Spatial Light Modulator. Sensors.

[14] Szeliski R. (2022). Computer Vision.

[15] Hung Y.Y., Lin L., Park B.G. (2000). Practical 3-D computer vision techniques for full-field surface measurement. Opt. Eng..

[16] Huang C.-n., Motavalli S. (1994). Reverse engineering of planar parts using machine vision. Comput. Ind. Eng..

[17] Alshennawy A.A., Gadelmawla E.S., Elewa I.M., Koura M.M. (2007). Construction of three-dimensional models of mechanical products from their orthographic views using computer vision. Proc. Inst. Mech. Eng. Part B J. Eng. Manuf..

[18] Mejia-Ugalde M., Dominguez-Gonzalez A., Trejo-Hernandez M., Morales-Hernandez L.A., Benitez-Rangel J.P. (2012). New approach for automatic tool selection in computer numerically controlled lathe by applying image processing. Proc. Inst. Mech. Eng. Part B J. Eng. Manuf..

[19] Mejia-Ugalde M., Trejo-Hernandez M., Dominguez-Gonzalez A., Osornio-Rios R.A., Benitez-Rangel J.P. (2013). Directional morphological approaches from image processing applied to automatic tool selection in computer numerical control milling machine. Proc. Inst. Mech. Eng. Part B J. Eng. Manuf..

[20] Gadelmawla E.S., Al-Mufadi F.A., Al-Aboodi A.S. (2014). Calculation of the machining time of cutting tools from captured images of machined parts using image texture features. Proc. Inst. Mech. Eng. Part B J. Eng. Manuf..

[21] Al-Kindi G., Zughaer H. (2012). An approach to improved CNC machining using vision-based system. Mater. Manuf. Process..

[22] Al-Kindi G., Zughaer H. (2011). Intelligent vision-based Computerized Numerically Controlled (CNC) machine. Lect. Notes Electr. Eng..

[23] Eladawi A.E., Gadelmawla E.S., Elewa I.M., Abdel-Shafy A.A. (2003). An application of computer vision for programming computer numerical control machines. Proc. Inst. Mech. Eng. Part B: J. Eng. Manuf..

[24] Al-Kindi G.A., Baul R.M., Gill K.F. (2007). An application of machine vision in the automated inspection of engineering surfaces. The Int. J. Prod. Res..

[25] Demircioglu P., Bogrekci I., Durakbasa N.M. (2013). Micro scale surface texture characterization of technical structures by computer vision. Measurement.

[26] Lawrence K D., Ramamoorthy B. (2013). Surface topography characterization of automotive cylinder liner surfaces using fractal methods. Appl. Surf. Sci..

[27] Worthington P.L., Hancock E.R. (2001). Surface topography using shape-from-shading. Pattern Recognit..

[28] Li H., Fang X., Zhu Z., Fu W., Zhao C. (2023). The approach of nanoscale vision-based measurement via diamond-machined surface topography. Measurement.

[29] He C.L., Zong W.J., Xue C.X., Sun T. (2018). An accurate 3D surface topography model for single-point diamond turning. Int. J. Mach. Tools Manuf..

[30] Zhao C., Cheung C.F., Liu M. (2019). Nanoscale measurement with pattern recognition of an ultra-precision diamond machined polar microstructure. Precis. Eng..

[31] Zhao C.Y., Cheung C.F., Fu W.P. (2021). An investigation of the cutting strategy for the machining of polar microstructures used in ultra-precision machining optical precision measurement. Micromachines.

[32] Yao S., Li H., Pang S., Zhu B., Zhang X., Fatikow S. (2021). A Review of Computer Microvision-Based Precision Motion Measurement: Principles, Characteristics, and Applications. IEEE Trans. Instrum. Meas..

[33] Fu W., Zhao C., Xue W., Li C. (2022). An investigation of the influence of microstructure surface topography on the imaging mechanism to explore super-resolution microstructure. Sci. Rep..

[34] Schonberger J.L., Frahm J.M. Structure-from-Motion Revisited. Proceedings of the 2016 IEEE Conference on Computer Vision and Pattern Recognition (CVPR).

[35] Liu H., Tang X., Shen S. (2020). Depth-map completion for large indoor scene reconstruction. Pattern Recognit..

[36] Gao X., Zhu L., Xie Z., Liu H., Shen S. (2021). Incremental Rotation Averaging. Int. J. Comput. Vis..

[37] Munkberg J., Chen W., Hasselgren J., Shen T., Müller T., Gao J., Fidler S. Extracting Triangular 3D Models, Materials, and Lighting from Images. Proceedings of the IEEE/CVF Conference on Computer Vision and Pattern Recognition, CVPR 2022.

[38] Fan B., Kong Q., Wang X., Xaing S., Pan C., Fua P. (2019). A performance evaluation of local features for image-based 3D reconstruction. IEEE Trans. Image Process..

[39] Leach R. (2011). Optical Measurement of Surface Topography.

[40] Batlle J., Mouaddib E., Salvi J. (1998). Recent progress in coded structured light as a technique to solve the correspondence problem: A survey. Pattern Recognit..

[41] Tian G.Y., Lu R.S., Gledhill D. (2007). Surface measurement using active vision and light scattering. Opt. Lasers Eng..

[42] Jain A., Gupta R. Gaussian filter threshold modulation for filtering flat and texture area of an image. Proceedings of the 2015 International Conference on Advances in Computer Engineering and Applications (ICACEA).

[43] Scarmana G. An application of the least squares plane fitting interpolation process to image reconstruction and enhancement: University of Southern Queensland Repository. Proceedings of the 78th FIG Working Week 2016: Recovering from Disaster.

[44] Kohn A.F. (2006). Autocorrelation and Cross-Correlation Methods.

[45] SciPy User Guide—SciPy v1.10.1 Manual. https://docs.scipy.org/doc/scipy/tutorial/index.html#user-guide.

[46] Signal Processing (scipy.signal)—SciPy v1.10.1 Manual. https://docs.scipy.org/doc/scipy/reference/signal.html.

[47] Statistical Functions (scipy.stats)—SciPy v1.10.1 Manual. https://docs.scipy.org/doc/scipy/reference/stats.html.

[48] Corder G.W., Foreman D.I. (2014). Nonparametric Statistics: A Step-By-Step Approach.

[49] Benesty J., Chen J., Huang Y., Cohen I. (2009). Pearson Correlation Coefficient. Noise Reduction in Speech Processing.

[50] Chai T., Draxler R.R. (2014). Root mean square error (RMSE) or mean absolute error (MAE)? -Arguments against avoiding RMSE in the literature. Geosci. Model Dev..

[51] Serda M., Balint G., Antala B., Carty C., Mabieme J.-M.A., Amar I.B., Kaplanova A. (2013). Synteza i Aktywność Biologiczna Nowych Analogów Tiosemikarbazonowych Chelatorów Żelaza.

[52] Kitasuka T., Aritsugi M., Rahutomo F. (2012). Semantic Cosine Similarity. https://www.researchgate.net/publication/262525676.

[53] Jekel C.F., Venter G., Venter M.P., Stander N., Haftka R.T. (2019). Similarity measures for identifying material parameters from hysteresis loops using inverse analysis. Int. J. Mater. Form..

[54] Bookstein A., Kulyukin V.A., Raita T. (2002). Generalized hamming distance. Inf. Retr. Boston..

[55] Theodoridis S., Koutroumbas K. (2009). Clustering: Basic Concepts. Pattern Recognition.

[56] pyxDamerauLevenshtein—PyPI. https://pypi.org/project/pyxDamerauLevenshtein/.

[57] similaritymeasures—PyPI. https://pypi.org/project/similaritymeasures/.

[58] Devogele T., Esnault M., Etienne L., Lardy F. Optimized Discrete Fréchet Distance between trajectories. Proceedings of the BigSpatial 2017—6th ACM SIGSPATIAL International Workshop on Analytics for Big Geospatial Data.

[59] Slavov S.D., Iliev I.V. (2016). Design and FEM static analysis of an instrument for surface plastic deformation of non-planar functional surfaces of machine parts. Fiability Durab..

[60] Nečas D., Klapetek P. (2012). Gwyddion: An open-source software for SPM data analysis. Cent. Eur. J. Phys..

[61] Minitab 21 Support—Minitab. https://support.minitab.com/en-us/minitab/21/.

[62] James G., Witten D., Hastie T., Tibshirani R. (2021). An Introduction to Statistical Learning.

